# The role of surgery for secondary mitral regurgitation and heart failure in the era of transcatheter mitral valve therapies

**DOI:** 10.31083/j.rcm2303087

**Published:** 2022-03-04

**Authors:** Pierre-Emmanuel Noly, Françis D. Pagani, Jean-Fançois Obadia, Denis Bouchard, Steven F. Bolling, Gorav Ailawadi, Paul C. Tang

**Affiliations:** 1 Department of Cardiac Surgery, University of Montreal, Montreal, QC H1T 1C8, Canada; 2 Department of Cardiac Surgery, University of Michigan, Ann Arbor, MI 48109-5864, USA; 3 Department of Cardiac Surgery, “Louis Pradel” Cardiologic Hospital, 69001 Lyon, France

**Keywords:** Secondary mitral regurgitation, Mitral valve replacement, Mitral valve repair, Heart failure, Left ventricular remodeling

## Abstract

The approach to the management of mitral valve (MV) disease and heart failure (HF) has dramatically changed over the last decades. It is well recognized that severe mitral regurgitation secondary to ischemic or non-ischemic cardiomyopathy is associated with an excess risk of mortality. Understanding the impact of the surgical treatment modality on mortality outcomes has been difficult due to the broad spectrum of secondary mitral regurgitation (SMR) phenotypes and lack of randomized surgical clinical trials. Over the last 30 years, surgeons have failed to provide compelling evidence to convince the medical community of the need to treat SMR in patients with severe HF. Therefore, the surgical treatment of SMR has never gained uniform acceptance as a significant option among patients suffering from SMR. Recent evidence from randomized trials in a non-surgical eligible patients treated with transcatheter therapies, has provided a new perspective on SMR treatment. Recently published European and American guidelines confirm the key role of percutaneous treatment of SMR and in parallel, these guidelines reinforce the role of mitral valve surgery in patients who require surgical revascularization. Complex mitral valve repair combining subvalvular apparatus repair along with annuloplasty seems to be a promising approach in selected patients in selected centers. Meanwhile, mitral valve replacement has become the preferred surgical strategy in most patients with advanced heart failure and severe LV remodeling or high risk of recurrent mitral regurgitation. In this comprehensive review, we aimed to discuss the role of mitral surgery for SMR in patients with heart failure in the contemporary era and to provide a practical approach for its surgical management.

## 1. Introduction

Secondary mitral regurgitation (SMR) is defined as a loss of mitral valve competency resulting from left ventricular (LV) or left atrial dysfunction and remodeling, in the absence of organic mitral valve disease. A recent epidemiologic analysis on a well identified American western population demonstrated that mitral regurgitation (MR) was the most frequent valvular disease with approximately, 1/3 of organic etiology, 1/3 ventricular SMR, and 1/3 atrial SMR [1]. Atrial SMR is a recently identified entity largely afflicting more females, older patients with better LV function, and less dilated ventricles. Its poor prognosis suggests that atrial SMR is possibly underexplored and possibly undertreated. In the absence of substantial literature about atrial SMR, this paper will focus on ventricular SMR.

The prevalence of moderate and severe SMR in the general population is estimated at 0.6%–1.2% and afflicts an estimated 2.6–5.2 million Europeans and 2.0–4.0 million Americans [2]. Among patients with heart failure with reduced ejection fraction (HFrEF), upwards of 25% of patients present with significant SMR [2,3]. Despite the improvement in medical management, SMR remains invariably and independently associated with an increased risk of hospitalization, death, and poor quality of life [3]. Moderate to severe SMR is associated with a two-fold increase in mortality when compared to patients with HFrEF with no or mild SMR, and a 20-fold increase in mortality compared to a sex and age-matched population [2].

Surgical approaches to correct SMR have been performed since the early 90s with a broad variety of techniques and outcomes [4]. The place of surgical treatment has always been a matter of controversy as reflected in recent studies failing to confirm its efficacy [5]. With the improvement of medical therapies, the development of percutaneous MV therapies, and a better knowledge of the results of surgical strategies in randomized trials, the place of surgical treatment for severe SMR has changed over time. The tremendous number of publications (>800) within the last 5 years and the publication of recent guidelines that include indications for TEER have prompted us to write this paper.

The purpose of this review is to discuss the pathophysiology of SMR, the place of medical management, surgical indications, and techniques for treatment of severe heart failure and SMR in the era of transcatheter mitral valve therapies. Transcatheter MV therapies are discussed in another article of this focus issue of the journal.

## 2. Understanding the pathophysiology of secondary mitral regurgitation

### 2.1 The atrio-ventriculo-mitral complex in SMR and heart failure

**Fig. 1.**
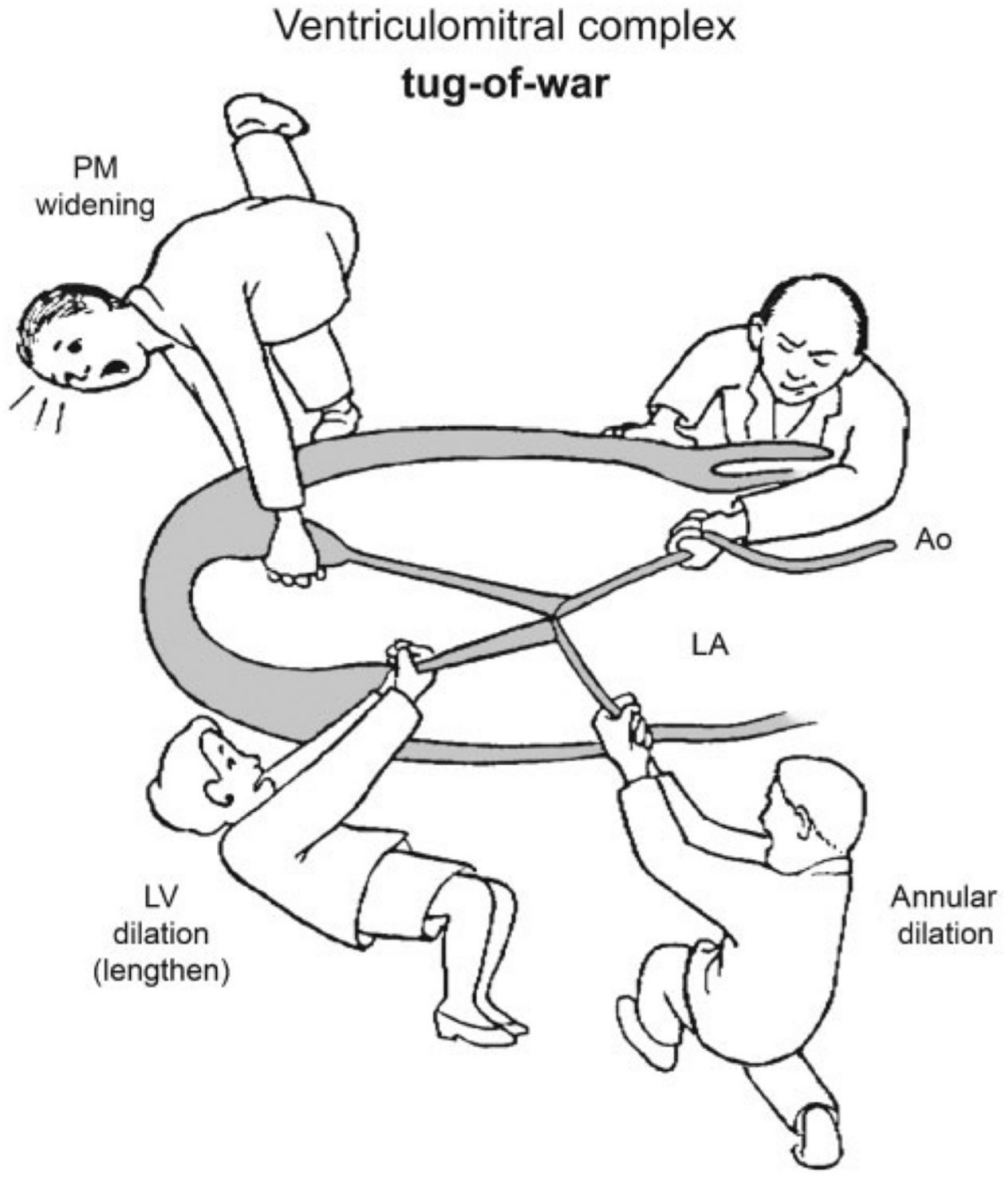
Mitral regurgitation in the setting of HFrEF is a direct marker of left ventricular (LV) impairment with two major consequences: (1) ventricular dilatation causing annular dilatation, reduction of the forces and the length of the coaptation of the anterior and posterior leaflets; (2) lateral displacement of the papillary muscle that induces tethering of the free edge of both leaflets. *Reproduced with permission from* C. Athanasuleas *et al*. [12].

Mechanisms leading to SMR have been extensively described [6-12]. They are dynamic, complex and involve all components of the mitral valvular and subvalvular apparatus. Mitral regurgitation in the setting of HFrEF is a direct marker of LV impairment with two major consequences: (1) ventricular dilatation causing annular dilatation, reduction of the forces and the length of the coaptation of the anterior and posterior leaflets; (2) lateral displacement of the papillary muscle that induces tethering of the free edge of both leaflets [7] (Fig. 1, Ref. [12]). Atrial dilatation leading to MV annular dilatation is a third important mechanism in the genesis of SMR [13,14]. This results in an increased static and pulsatile load on the left atrium and the pulmonary circulation. In addition, the regurgitant volume reduces the effective LV stroke volume, and results in an increase of end-diastolic volume, and potentially increased LV wall stress and myocardial oxygen consumption, contributing to a vicious circle of worsening myocardial impairment and MR severity [15]. Thus, MR has a detrimental effect on both the left and right ventricle.

### 2.2 Identify the phenotype of SMR

Understanding the phenotype of SMR is a key step to tailor the surgical approach and provide the best outcomes. Even though they share some pathophysiological mechanisms, ischemic and non-ischemic cardiomyopathy may be associated with two distinct phenotypes of LV remodeling (asymmetrical and symmetrical remodeling). SMR phenotype is impacted by many factors related to the extent of myocardial impairment: (1) the degree of global or regional LV dysfunction and remodeling, (2) the degree of LV and MV annulus dilatation, (3) the presence or extension of LV scar, (4) the viability of the postero-lateral wall, (5) the degree of papillary muscles (PM) desynchrony, (6) the degree of posterior leaflet tethering, and (7) the compensating elongation of the MV leaflets.

In asymmetrical SMR, the posterior leaflet is moved more posteriorly than apically, and its tethering leads to a pseudo-prolapsus of the anterior leaflet creating an eccentric mitral regurgitant jet. After myocardial injury (scare, myocardial stunning, hibernation secondary to ischemia) in the papillary muscle (PM) territories, the PM lose their primary function and the surface of the mitral annulus increases. Annular dilatation develops especially in the septal-lateral direction, as well as asymmetric dilation at the P3 segment, leading to the asymmetrical MR phenotype [8,16]. The loss of the normally elliptical and the saddle-shape of the annulus might contribute to the increased stress on the leaflets and compromise their coaptation.

In the symmetric phenotype, impairment of the myocardium is global, leading to a spherical-shape remodeling and annular mitral valve dilatation. The equal apical and mediolateral displacement of the PM leads to a systolic restriction of both anterior and posterior leaflets and a more pronounced displacement of the coaptation point [4]. The regurgitant jet is usually central and reflects the symmetrical tethering forces in both leaflets. The presence of left bundle branch block can exacerbate SMR due to reduced closing forces and dyssynchronous PM contraction [ 17,18]. In those situations, resynchronization of the LV can significantly reduce the MV regurgitation.

Assessment of the characteristics of the subjacent myocardium in SMR is also of major importance in the management of SMR. First, LV scar quantified by late gadolinium enhancement may alter the prognosis of patients with MR secondary to ischemic and non-ischemic cardiomyopathy [19]. The natural history of SMR might be more impacted by the burden of scar, rather than the degree of SMR. In patients with extensive scar burden, the prognosis might be driven by the scar burden and the underlying cardiomyopathy rather than the degree of MR itself [20]. Secondly, reverse remodeling after mitral valve repair may be less likely to occur in presence of scar or severe leaflet tethering which is associated with a higher rate of recurrent MR. Thus, the extent of scaring of the left ventricle may impact the probability to reliably repair SMR and influence the decision to perform replacement of the MV over that of repair.

## 3. Management of SMR and HFrEF

### 3.1 The importance of the guideline-directed medical therapy

GDMT including cardiac resynchronization therapy (CRT) and myocardial revascularization should be the first line of treatment for a patient with heart failure and SMR, regardless of the LVEF [21-25]. By reducing LV afterload, decreasing LV remodeling, increasing LV contractility, and improving PM synchronism, medical treatment can reduce the regurgitant volume. Importantly, we learned from the MITRA-FR trial and the COAPT trial that MR can decrease from 32.5% to 46.9% in patients treated with GDMT alone, as highlighted in the control group of the two studies. This was unexpected since, in both studies the patients were supposed to be on optimal medical management and stable for 3 months prior to randomization. This suggests that in the real world, GDMT is likely not optimized in a significant proportion of patients and the Class I recommendation from both ESC/EACTS and ACC/AHA for optimization of GDMT is largely not met.

Among patients with HFrEF, GDMT is associated with an improvement of SMR severity (≥1-grade reduction) in 27% to 68% of patients evaluated for possible MV intervention or who received MT only [26,27]. The Pharmacological Reduction of Functional, Ischemic Mitral Regurgitation (PRIME) study suggested that the angiotensin receptor neprilysin inhibitor (ARNI), sacubitril/valsartan might be more effective than an angiotensin receptor blocker alone (valsartan) to improve SMR in HFrEF patients [28]. In this double-blind, randomized trial including 118 patients with HF and severe LV dysfunction (LVEF = 34%), sacubitril/valsartan resulted in a greater reduction in effective regurgitant orifice area (EROA), regurgitant volume, and LVEDV index at 1-year follow-up. However, the majority of patients had only mild-to-moderate SMR and only 5% of the patients had severe SMR at baseline.

Thus, GMDT alone is not sufficient in more than 50% of patients, especially if SMR is severe and there is advanced LV remodeling. These “non-responders” have a sustained severe SMR or worsening of SMR and have a poor prognosis [27]. They usually present with more advanced disease, frequently receive higher doses of loop diuretics, aldosterone antagonists, and less frequently receive angiotensin-converting enzyme inhibitors or angiotensin receptor blockers alone or combined with ARNI. A higher EROA/LVEDV ratio, a longer QRS, and a left bundle branch block have been proposed as predictors of a negative response to GDMT [26,27]. This was observed in a secondary analysis of the COAPT trial but not confirmed in sub-group analysis of MITRA.FR trial [29].

Cardiac resynchronization therapy improves survival in patients with HFrEF and prolonged QRS secondary to ischemic and non-ischemic cardiomyopathy [23,30]. By restoring a synchronous LV and PM contraction and by reducing leaflet tethering and closing forces on the MV apparatus, CRT can improve MV function. Studies have shown that CRT results in a sustained reduction in the severity of SMR from 50% to 70% [18,27,31-33]. In a cohort of 1313 patients treated with CRT, 26% had moderate to severe SMR which remained unchanged at 6 months [18]. This subgroup of patients had an increased risk of death, independently of the clinical and LV volumetric responses to CRT. Positive predictors of MR reduction included an LV end-systolic dimension index <29 mm/m^2^, absence of scar at the point of papillary muscle insertion, and anteroseptal to posterior wall radial strain dyssynchrony >200 ms [34]. Older age, longer QRS duration, and septo-lateral delay were independent predictors of MR improvement after CRT [32].

### 3.2 Surgical treatment of SMR

Although the prognosis of SMR patients who do not respond to GDMT, coronary revascularization and CRT is poor, the benefit of the surgical treatment on survival has never been established. The differences in patients’ characteristics, the heterogeneity of phenotype of SMR, the lack of general agreement regarding the definition of SMR severity, and the diversity of the surgical techniques may contribute to the lack of strong evidence despite a growing wealth of literature. Only one randomized trial demonstrated effectiveness of MV repair combined to surgical revascularization in improving functional capacity, left ventricular reverse remodeling, MR severity, and B-type natriuretic peptide levels (*Randomized Ischemic Mitral Evaluation* (RIME trial)) [35]. The high rate of MR recurrence after annuloplasty alone (up to 58% at 2 years in the CTSN trial [36]) is concerning and has led many surgeons to switch from repair to replacement of MV in the setting of SMR and HF. Targeting heart failure patients who will benefit the most from mitral valve surgical intervention is key. The role of surgery for SMR has evolved in the European and American guidelines over time [5], and in the next paragraphs we will focus on the most recent European and American guidelines and expert consensus [21-25,37].

#### 3.2.1 What are the latest recommendations for surgery in SMR?

Until the publication of the 2021 ESC/EACTS guidelines, the definition of severe SMR was different between US and European guidelines [21,24]. European guidelines defined severe SMR with lower EROA and lower regurgitant volume (20 mm^2^ versus 40 mm^2^, and ≥30 mL versus ≥60 mL) and those different criteria perhaps, explain the different inclusion criteria between MITRA.FR and COAPT clinical trials. Mitral valve hemodynamic criteria to define severe SMR are now similar to primary MR in both current European and American guidelines [21,22]. Criteria for severe SMR are met if the EROA is measured ≥40 mm^2^, the regurgitant volume is ≥60 mL, and the regurgitant fraction is ≥50%, in absence of structural anomaly of the mitral valve. European recommendations extend the criteria towards a lower EROA threshold to account for the crescent shape of the regurgitant orifice, allowing characterization of severe SMR in presence of an EROA ≥30 mm^2^. It has been suggested that this definition of secondary MR, should be revisited and should consider the LV geometry and myocardial enviromnent [5,38].

There has been no randomized trial comparing GDMT + surgery versus GDMT alone in patients with nonischemic cardiomyopathy and HRrEF with SMR. In patients with HFrEF and significant SMR secondary to ischemic cardiomyopathy, 5 randomized trials failed or were not powered to demonstrate a survival benefit from concomitant mitral valve surgery at the time of surgical revascularization [35,36,39-41]. Therefore, indications for surgical treatment of SMR are limited and mitral valve surgery is only recommended in patients who remain symptomatic despite optimal medical treatment when a percutaneous procedure is not possible. Thus, very few patients would be considered for surgery except in the absence of a concomitant indication for coronary revascularization. In absence of strong evidence that surgical intervention for SMR provides a survival benefit, most of the guideline recommendations remain graded at class II with a level of evidence (LOE) at B or C. Indications for MV surgery with or without concomitant coronary artery bypass grafting (CABG) are summarized in Table 1 (Ref. [21-25,37]).

The 2021 ESC/EATCS guidelines [21] for valvular heart disease and the AATS consensus for CABG in ischemic cardiomyopathy with heart failure [37], recommend concomitant mitral surgery (Class I; LOE, B, and B-R) for patients with severe SMR and who need cardiac surgery for other reasons, coronary revascularization being the most frequent. The ACC/AHA 2020 guidelines for valvular heart disease and the ESC 2021 guidelines for heart failure graded this recommendation Class IIa; LOE, B-NR, and C, respectively. Concomitant MV surgery may be considered in selected patients with ischemic cardiomyopathy and moderate SMR (Class IIb; LOE, B-NR). Factors influencing surgical treatment are: (1) the presence of both viability and ischemia in the posterolateral wall; (2) graftability of posterolateral coronary artery targets; (3) presence of atrial arrhythmias, left atrial dilatation, organic mitral valve disease, and/or severe left ventricular dilatation; and (4) heart failure symptoms predominate. In both US and European guideline recommendations, isolated MV surgery may be considered in symptomatic patients with HFrEF and SMR secondary to non-ischemic cardiopathy, if they are judged appropriate for surgery by the heart team (Class IIb; LOE, C). Recommendations for the type of intervention (repair, replacement, LV reconstruction) vary substantially between societies. According to the ACC/AHA 2020 guidelines, it may be reasonable to choose chordal-sparing mitral valve replacement over downsized annuloplasty, without specifying selection criteria (Class IIb, Level B-R). In the 2017 ACC expert consensus, MV replacement may be the primary approach in a patient with severe LV remodeling, annular dilatation with severe leaflet tethering, or presence of an infero-basal aneurysm. Conversely, the 2021 ESC/EATCS guidelines and the 2017 ACC expert consensus favored MV repair whenever feasible or in selected patients in conjunction with secondary or tertiary chordal cutting or other adjunctive procedures.

#### 3.2.2 Patient selection for surgical mitral intervention

Patients with severe SMR and HFrEF who remain symptomatic despite GDMT including CRT must be referred early to a multidisciplinary heart team including a heart failure specialist, interventional cardiologist dedicated to structural heart disease, cardiac surgeon with mitral valve disease expertise, electrophysiologist, and anesthesiologist.

In a recent population-based study of 13,223 patients who meet the eligibility criteria for heart failure according to guideline definition, the overall prevalence of severe SMR was 10%, and was 25% in patients with HFrEF [2]. Compared to mild and moderate MR, severe SMR was associated with a more pronounced ventricular component (larger LVEDD) and a greater prevalence of ischemic heart disease. Despite accessibility to state-of-the-art healthcare facilities, MV repair or replacement was performed only in 1.5% and 1.1% of patients with moderate-to-severe and severe SMR. In the subgroup of patients with HFrEF (LVEF <50%), 3.1% underwent a mitral intervention [2]. According to the STS database, isolated MV surgery for NICM and ICM represents 2.9% and 1.3% of all MV surgery performed [42]. This conservative approach might be partially explained by the lack of evidence that surgical MV repair or replacement can improve survival in SMR and the excessive risks associated with surgical treatment in an elderly and sick population. Most of the patients assessed had associated significant comorbidities that might preclude the surgery (hypertension 61%, CAD 50%, diabetes 26%, atrial fibrillation 30%, cerebral vascular disease 18%, and peripheral vascular disease 24%). In all randomized-controlled trial assessing MV surgery for SMR, patients with papillary muscle rupture, recent myocardial infarction, severe comorbidities such chronic kidney failure, liver disease, life expectancy <12 months, LVEF <30%, or a previous surgery were excluded (Table 2, Ref. [35,39-41,43,44]).

The decision-making process for surgical treatment is based on an exhaustive workup including a thorough clinical assessment for symptoms, health-related quality of life, signs of heart failure, comorbidities, echocardiographic imaging at rest and at stress, coronary angiography, and medications (Table 3). A systematic stepwise approach for surgical consideration in patients with SMR and could be summarized as follow [4,24,45,46]:

(1) Confirm the severity of SMR according to the most recent guidelines.

(2) Confirm that the patient receives GDMT including angioplasty and CRT.

(3) Confirm that the patient remains symptomatic and that the symptoms are related to MR.

(4) Evaluate surgical risk.

(5) Rule out end-stage heart failure with a thorough hemodynamic evaluation.

(6) Assess the need/possibility for coronary revascularization or other cardiac surgery.

(7) Assess mitral valve anatomy, and the degree of LV remodeling (geometry of the left ventricle), and myocardial characteristics (viability, scar, aneurysm, fibrosis) (Table 3).

(8) Determine the risk of recurrence of MR after surgical repair. Factors associated surgical repair failure are: long duration of Cardiac Heart Failure, LVEDD >65 mm, LVESD >51 mm, tenting Height >10 mm, posterior Leaflet-annulus angle >45°, distal ant leaflet-annulus angle >25°, end Syst interpapillary distance >20 mm, systolic sphericity index >0.7, symmetric LV instead of normal, asymmetric LV shape [4,47-51].

(9) In case of unfavorable anatomy for a TEER and high surgical risk, discuss percutaneous or apical approach for MV replacement.

The optimal candidate for MV surgery for SMR would be a patient with chronic heart failure with symptoms despite GDMT and CRT, with reduced LVEF and severe SMR associated with CAD amenable to surgical revascularization, especially in the viable latero-posterior wall, and if the patient is deemed to be low to intermediate surgical risk by a multidisciplinary team. In absence of CAD, surgery is usually indicated in low surgical risk patients with nonsuitable anatomy for TEER. MV repair may be the primary approach in absence of prognostic factors for MR recurrence and in experienced centers, and chordae-sparing mitral valve replacement in other cases. According to these criteria, the proportion of SMR patients fulfilling these requirements is probably low.

#### 3.2.3 How to tailor the surgical approach for SMR in HFrEF?

By restoring MV competency, MV surgery stops the vicious cycle induced by the regurgitant volume, reduces the left ventricular volume overload, and potentially reverses LV remodeling and improves LV function. Unlike non-surgical therapies, surgical MV repair or replacement allows correction of SMR at the subvalvular and valvular level. Preservation of the subvalvular apparatus is essential to achieve good outcomes and to reduce long-term mortality [52,53], especially after MV replacement in patients with severe LV dysfunction. In the setting of SMR, leaflets are rarely calcified and both anterior and posterior subvalvular apparatus can be spared. GorTex sutures might be used to relocalize the PM posteriorly and laterally and eventually avoid prosthesis leaflet obstruction. In case of repair, the principal goals are to restore leaflet coaptation depth to >5 mm, stabilize and remodel the annulus, restore normal leaflet motion, and ultimately eradicate MR permanently. Surgeon experience has been recognized as a primary determinant of successful repair in both primary MR and SMR. As discussed earlier, assessment of the MR phenotype is a key step before considering an MV repair in the setting of SMR.

##### 3.2.3.1 Patients with severe ischemic cardiomyopathy and severe SMR.

In patients with ischemic cardiomyopathy and significant SMR who are good candidates for surgical revascularization, MV repair and MV replacement are both valid options. The Cardiothoracic Surgical Trials Network (CTSN) randomized trial compared restrictive annuloplasty and chordal-sparing replacement in patients with severe ischemic SMR [43]. At a 2-year follow-up, there was no difference in the extent of LV reverse remodeling, LVEF, or death [36]. However, the rate of significant recurrent MR (moderate or severe) was significantly higher after MV annuloplasty (58% versus 3.8%). Compared to older studies that have generally demonstrated greater mortality with MV replacement versus repair, the CTSN study demonstrated that chordal-sparing MV replacement is a safe and valid option for severe SMR secondary to ischemic cardiomyopathy. Subsequently to these results and because it requires less technical skills, many *non-mitral* surgeons tend to favor MV replacement over MV repair in these patients. Even though we are waiting for the long-term results of comparison between MV repair and replacement, we may hypothesize that the durability of the prosthesis exceeds the life expectancy of these critically hill patients.

**Fig. 2.**
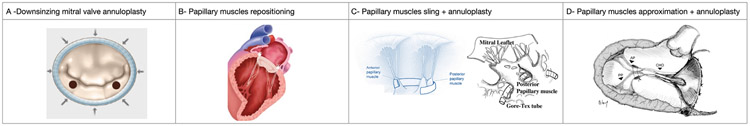
Surgical techniques for mitral valve repair in secondary mitral regurgitation. (A) Downsing mitral valve annuloplasty. *Reproduced with permission from J. Yap et al.* [59]. (B) Papillary muscles repositioning + annuloplasty. PM realignment sutures are through the posteromedial papillary muscle and through the posterior mitral annulus in the P3 segment. *Reproduced with permission from E. Girdauskas et al.* [54]. (C) Papillary muscles sling using a 4 mm Gore-Tex tube encircling the bodies of posteromedial and anterolateral papillary muscles. *Reproduced with permission from F. Nappi et al.* [60] *and U. Hvass et al.* [58]. (D) Papillary muscle approximation. A U shaped 2-0 Gore-Tex suture reinforced by two patches of autologous pericardium is passed through the bodies of the posterior and anterior papillary muscles. *Reproduced with permission from A. Rama et al.* [57].

The risk of recunent MR, “the Achille Heel” of MV interventions, and included in any publication reporting surgical MV repair is surprisingly not present as an outcome in the COAPT Trial. After 2 years of followup, patients in the Mitraclip arm of the COAPT trial remained stable with no recurrent MR. Even the 17% of recurrent MR seen in the Mitraclip arm of MITRA.FR is less than the rate usually reported after surgical MV repair. This suggest that the downsizing annuloplasty is possibly not the best option to cure SMR and reinforces the necessity to act not only on the annulus but also on the sub-valvular apparatus (leaflets, Papillary muscles). Several techniques of MV repair associated with PM interventions have been described [54-58] (Fig. 2, Ref. [54,57-60]). A meta-analysis including one randomized controlled trial and four propensity-matched studies showed greater LV reverse remodeling and systolic function, 57% reduced risk of recurrence of moderate or greater MR, and an improved geometry of the MV apparatus at short and mid-term follow-up when MV annuloplasty is performed with subvalvular MV repair [56]. PM relocation seems as efficient a method to repair MR secondary to ischemic and non-ischemic cardiomyopathy [61]. In patients undergoing surgical heart failure therapy for ischemic cardiomyopathy (ICM), the extent of basal fibrosis characterized by MRI might be a useful predictor of postoperative LV systolic and diastolic functional recovery and postoperative adverse outcomes [62].

However, many questions remain. First, how do we identify responders following MV repair? Interestingly, in a subgroup of patients of the CTSN trial, MV repair was associated with a significant improvement of LV remodeling (decrease of LVESI by >15%) and no recurrent MR. A sub-analysis of these “responders” might have been useful to determine if these patients had MR characterized as the disproportionate type. Second, the technique for MV repair was left at the surgeon’s discretion and most of the patients underwent undersized annuloplasty alone. Knowing that subvalvular repair combined with MV annuloplasty might improve LV remodeling and decrease the rate of recurrent MR [54], we may wonder if a better standardization of MV repair in SMR may improve outcomes.

##### 3.2.3.2 Patients with severe non-ischemic cardiomyopathy and severe SMR.

In this population, we cannot count on the beneficial effect of myocardial revascularization to contribute to improving LV remodeling. A thorough hemodynamic evaluation in patients with severe SMR and severe HfrEF is of paramount importance. In a recent study, Kashiyama *et al.* [63] found that an LV stroke work index <25.9 g-m/m^2^/beat was strongly associated with allcause mortality, admission for heart failure, or left ventricular assist device implantation after MV surgery. In this series including 53 patients with non-ischemic cardiomyopathy (NICM) and severe HfrEF (LVEF <35%), and advanced ventricular remodeling (LVEDD >75 mm, LVESD >67 mm, freedom from composite outcomes including allcause mortality or admission for HF/LVAD implantation was 58.0% at 1 year. 38% of them had prophylactic IABP after surgery, 20% died from cardiac failure and 17% had an LVAD within the year following surgery. Although the number of patients is small, this study provides an appealing decisional algorithm for strategy in patients with severe NICM and SMR. Since functional MR itself accelerates the progression of LV remodeling via volume overload, MV surgery could delay or arrest the progression of LV remodeling or clinical symptoms. The superiority for MV repair over replacement in this population is not established. For a patient with severe remodeling, replacement might be the first option. Patients with severe SMR and LVEF <35% have a higher degree of LV remodeling and prognostic factors for recurrence of MR after MV repair. In this category of patients, freedom from recurrent MR is longer after MV replacement than after MV repair. The excess of mortality after MV replacement compared to MV repair is likely related to more advanced disease and a higher rate of comorbidities in This population. Finally, as the outcomes after durable left ventricular assist devices improve, we may wonder if some patients would benefit most from such therapy compared to MV surgery.

In patients with severe SMR and very low LVEF (<25%), preoperative optimization is extremely important to ensure euvolemia, good end-organ perfusion, and function. Preemptive temporary mechanical circulatory support with intra-aortic balloon pump (IABP) or another device might be considered either before surgery or after weaning cardiopulmonary bypass weaning [64]. The failing LV has then submitted simultaneously to the stress of the cardiac arrest and cardiopulmonary bypass and an increase of afterload. Inotropic support, afterload reduction, volume management are mandatory to reduce the risk of postoperative death and low cardiac output. Again, temporary mechanical circulatory support might be necessary to unload the left ventricle and provide appropriate systemic perfusion while waiting for myocardial remodeling and recovery.

An observational, prospective international study on surgical treatment of secondary mitral regurgitation (The SMR study) aims to understand which is the technique, or the strategy, more efficient to treat SMR and to assess the real efficacy of the surgical treatment [65]. By describing the surgical practice over 5 years, the authors will help to better evaluate the risk factors for a worse result (death, re-hospitalization for heart failure, reoperation for MR return, moderate, or more MR return).

## 4. Transcatheter edge-to-edge repair or MV surgery?

There is no doubt that TEER with the MitraClip system is a very safe option with very few complications even in SMR patients with poor LV function. TEER is often presented as complementary to surgery [21,66-68] which is probably idealistic if we consider that the proportion of patients with SMR referred to surgery has never been significant. In a recent survey, only 3% of patients with SMR were referred to surgery [1]. To date, TEER is indicated to treat severe symptomatic MR in patients who have prohibitive surgical risk, and favorable anatomy after a multidisciplinary team evaluation [21]. Despite initial concerns regarding the impact of the growth of TEER on mitral surgical practice, implementation of a TEER program is usually associated with a “halo effect” and a growth of approximately 10% in annual surgical volume for MV disease [69-71]. Among patients referred to a multidisciplinary team for TEER assessment, only the third had the procedure [72,73] and among patients denied for TEER, almost 20% are deemed good candidates for surgery [70,73]. Furthermore, the introduction of TEER did not have an impact on the clinical characteristics and operative risk of patients who underwent surgical MV intervention which are mainly proposed in addition to coronary revascularization. Several studies suggest benefits of complementary procedures for all treated MR patients and that introduction of a TEER program may be associated with favorable clinical outcomes.

Although 27% of patients included in the landmark randomized trial EVEREST II had SMR [74,75], there has been no dedicated randomized trial comparing TEER to surgery in patients with HfrEF and SMR. Two recent retrospective studies compared the efficacy and clinical outcomes between TEER and surgery among patients with SMR and HfrEF using a propensity-matched analysis [76, 77]. Okuno *el al.* [76] compared the midterm clinical outcomes in the 2 years between patients undergoing TEER with the Mitraclip system and patients undergoing surgical MV repair using undersized ring annuloplasty (n = 202). In the unmatched cohort, patients who underwent surgical repair were younger, with a lower surgical risk, and had lower MR severity. After propensity-score matching, the rate of previous coronary stenting and severe MR were higher in the TEER group. Although the rate at 2 years of moderate and severe MR (40% versus 13.5%, *p* < 0.001), and the rate of MR progression were higher in the TEER group, all-cause death did not differ between the 2 matched groups (24.3% versus 23%, *p* = 0.909). Improvement of LVEF was observed only in the surgical group. In the second study, Gyoten *et al.* [77] included 132 patients with SMR and LVEF ≤30% treated either with surgical MV repair (n = 47) or with MitraClip (n = 85). Again, patients who underwent TEER were older, had a higher surgical risk, and had a more advanced HF as reflected by lower LVEF, higher LV volumes, and diameters. TEER resulted in lower perioperative complications and mortality than surgical therapy but yielded less reduction in SMR which is consistent with the EVEREST II trial and the study from Gyoten *et al.* [77]. After propensity-score matching for age, logistic EuroSCORE, and LVESV, freedom from cardiac death at 1 and 3 years, and freedom from re-hospitalization at 1 were higher after surgical MV repair.

## 5. How to integrate the findings of the MITRA.FR and the COAPT trials in surgical candidates?

Even though the natural history and pathophysiology of SMR is well appreciated, the controversy surrounding the conflicting results between (MITRA.FR [66,68] and COAPT [67,78]) provide a great opportunity to better understand the physiopathology of SMR, patient selection and outcome with intervention. First introduced by Grayburn *et al.* [79], the concept of “proportionate” and “disproportionate” MR tried to reconcile the findings of these two trials. This concept consists of adjusting the effective regurgitant orifice area (EROA) cut-off to the left ventricular end-diastolic volume (LVEDV) and the LVEF [6,79-81]. In proportionate MR, the degree of MR is as expected to the degree of LV dilatation and dysfunction. In other words, proportionate SMR is more a ventricular disease than a MV disease and the main cause of regurgitation is the dilatation and symmetrical tethering of the mitral valve leaflets due to LV enlargement. In disproportionate SMR, the degree of MR is larger than expected to the degree of LV dilatation and dysfunction. MR is typically associated with a focal LV wall motion abnormality, leading to an asymmetrical regurgitant jet and a degree of MR that cannot be ascribed to global LV dilatation. This seducing concept supported by the larger LV volumes reported in COAPT versus those in MITRA.FR is still debated. It is now clear that the difference in the LV volumes is mainly due to different evaluation of the core labs of the two studies. Actually, in a secondary analysis of the MITRA.FR patients, there was no difference between patients with proportionate and disproportionate MR [82]. To support the prognostic impact of This concept, Lopes and al. developed a method of assessing the proportionality of SMR using a simplified equation integrating LVEF, regurgitant fraction, and LVEDV [83]. An individualized theoretical regurgitant volume threshold was calculated, above which disproportionate SMR will be present (individualized theoretical regurgitant volume =50% × LVEF × LVEDV). If the measured regurgitant volume is above 7.7 mL of the theoretical regurgitant volume, the SMR was classified as disproportionate. The authors showed that patients with proportionate and disproportionate MR are two distinct populations, the later having a more advanced clinical condition and heart failure, and a worst prognosis. Interestingly, the SMR proportionality concept showed greater discriminative power to predict allcause mortality than the AHA/ACC and ESC classification guidelines. Of note, during the 3.8 years of the study period, only 16 (2.7%) patients underwent TEER, 6 (1%) underwent mitral valve replacement or repair, and 11 (1.9%) patients received a heart transplant or an LVAD. This concept of proportionate and disproportionate MR may help us to identify patients with the worst prognosis and in whom the mitral valve dysfunction plays a dominant role over the LV dysfunction. In such patients, MV repair may prove a better opportunity to achieve a durable result.

This theory has been challenged by a recent study from the Mayo Clinic [84]. In a cohort of 6381 HfrEF patients with SMR medically managed, EROA was a strong predictor of excess mortality, independent of other LV parameters. Compared to the general population, the authors found that excess mortality started at low EROA (10 mm^2^) and became considerable before the established threshold (40 mm^2^) in US and European guidelines, requestioning the current grading system for SMR. Furthermore, the excess mortality was more pronounced when compared to patients with degenerative MV disease. Patients with severe SMR represented 12% (EROA ≥30 mm^2^), and 8% (EROA ≥40 mm^2^) and faced the highest excess of mortality with a 2-year survival of 29%, which is far less than the control groups in MITRA.FR and COAPT.

How should we integrate these findings for the surgical approach of SMR in HfrEF? In absence of a new trial assessing the role of surgery for SMR, the responses are purely speculative leading to propose an integrative approach including multiple parameters. Multi-vessel coronary artery disease with confirmed viability leads to a preference for surgery when severe SMR needs to be corrected (EROA >0.3 mm^2^, LVEF 20–50%, LVESD <70 mm, systolic pulmonary pressure <70 mmHg). In these patients, MV repair may provide the best results in those patients with favorable anatomy; (1) tenting <10 mm; (2) annulus to post leaflet angle <45°; (3) sphericity index >0.7; and (4) inter-papillary muscle distance <20 mm. Otherwise replacement should be preferred with sub-valvular apparatus preservation [49]. For patients with severe HfrEF and in which SMR is likely proportionate, advanced heart failure therapies may be the most appropriate.

## 6. Conclusions

Heterogeneity of the pathology underlying SMR and the variability of its definition make SMR a moving target for clinical research. In absence of new evidence for survival benefit, the place of MV surgery in patients with SMR and HF has not changed over the last few years and remains limited. Chordal-sparing MV replacement has become the first-line surgical strategy for many surgeons, especially in cases of severe LV remodeling or when the risk of recurrent MR after repair remains high. Half of the patients with significant SMR and heart failure fail to improve after optimization of GDMT including CRT and new heart failure therapies. New insights from studies comparing TEER to medical treatment has proven that in highly selected patients the correction of SMR have a dramatic positive impact. As a consequence, and taking into account its safety, TEER has been largely adopted worldwide for treatment of SMR. This growing interest for SMR could be associated with an increase of surgical volume (halo effect).

After numerous RCTs confirming the success story of TAVI’s, we are today expecting more RCT studies to evaluate the place of TEER, TMVR and surgery for SMR (>80 registered on Clinical-trial.gov). The tremendous investment of the industry in This field is expected as SMR remains a public health problem that needs to be solved. The development and the spread of new less invasive MV interventions will help to fill the gap between medical treatment and surgery.

## Figures and Tables

**Table 1.** Summary of the indications for therapies in SMR and HF according to recent guidelines and scientific statements.

Guidelines	ACC/AHA guidelines — valvular heart disease 2020 [22]	ESC guidelines — valvular heart disease 2021 [21]	ESC guidelines — heart failure 2021 [23]	AATS consensus — CABG in ischemic cardiomyopathy and heart failure 2021 [37]	Join statement HFA/EACVI/EHRA/EAPCI — Mitral valve therapies 2021 [24]	ACC expert consensus for MR 2017 [25]
Type of procedure						
Isolated Mitral valve Surgery	Class IIb, level B-NR Patient with severe SMR and LVEF <50%, who has persistent symptoms while on optimal GDMT	Class IIb, Level C Symptomatic patients with severe SMR judged appropriate for surgery by the Heart Team	Class IIb, Level C Patients with moderate-to-severe or severe SMR, still symptomatic despite GDMT and at low surgical risk	No statement	No statement	No statement
Mitral valve surgery and surgical revascularization	Class IIa, level B-NR	Class I, Level B	Class IIa, Level C	Class I, level B-NR	Patient with moderate/severe SMR, symptomatic while on optimized GDMT and CRT	No statement
	Patient with severe SMR (stages C and D) when CABG is undertaken for the treatment of myocardial ischemia	Patients with severe SMR, symptomatic despite optimal GDMT (CRT included), undergoing CABG or any other cardiac operation	Patients with severe SMR and CAD who need revascularization	Patient undergoing CABG with severe SMR Class IIb, Level B-NR Patient undergoing CABG with moderate SMR	And undergoing CABG with a low surgical risk	
Surgical consideration	Class IIb, Level B-R	Repair whenever possible	No statement	Class IIa, B-R	No statement	**Restrictive remodeling rigid annuloplasty ring** May be used as a primary modality for annular dilatation mechanism
	Chordal-sparing mitral valve replacement may be reasonable to choose over downsized annuloplasty			Concomitant surgical ventricular restoration should be considered for patients with a true left ventricular aneurysm.		It May be used in conjunction with secondary or tertiary chordal cutting
						It May be used with other adjunctive procedures
						Should be avoided as sole therapy in the setting of Carpentier Type IIIB mechanism with left ventricular inferobasal aneurysm
						**Chord-sparing mitral valve replacement** may be used as a primary modality for annular dilatation with severe leaflet tethering (i.e., >10 mm tenting height) or presence of inferobasal aneurysm
Transcatheter edge-to-edge repair	Class IIa, Level B-R	Class IIa, Level C	Class IIa, Level B	No statement	HF with moderate/severe MR persistent symptoms despite GDMT optimization	No statement
	Patients with persistent symptoms, while on optimal GDMT (stage D), with appropriate anatomy as defined on TEE and who meet COAPT criteria*	In symptomatic patients, who are judged not appropriate for surgery by the Heart Team based on their characteristics, PCI (and/or TAVI) possibly followed by TEER (in case of persisting severe SMR). Class IIa, Level B	Patients symptomatic despite GDMT not eligible for surgery and not needing coronary revascularization who fulfill criteria for achieving a reduction in HF hospitalizations (COAPT criteria inclusion) *		No end-stage HF	
		Selected symptomatic patients, not eligible for surgery and fulfilling criteria suggesting an increased chance of responding to the treatment			With COAPT criteria*	
		Class IIb, Level C	Class IIb, Level C		If CAD, associated with PCI in high-risk patients	
		In high-risk symptomatic patients not eligible for surgery and not fulfilling the criteria suggesting an increased chance of responding to TEER, the Heart Team may consider in selected cases a TEER procedure or other transcatheter valve therapy if applicable, after careful evaluation for ventricular assist device or heart transplant	To improve symptoms in patients highly symptomatic despite GDMT, not eligible for surgery and not needing coronary revascularization, and who do not fulfill COAPT criteria			
Advance HF therapies (HT, VAD)	No statement	No statement	No statement	Class IIa, Level B-NR	LVAD or HT should be considered in patients with end-stage heart failure.	No statement
				Can be considered as alternatives to CABG for patients in NYHA functional class IV who have predictors of poor heart failure survival Class IIa, Level C-LD Advanced surgical therapies such as LVAD insertion or heart transplantation can be considered as alternatives to CABG for patients in NYHA functional class IV who are anatomically high risk for CABG		

ACC, American College of Cardiology; AHA, American Heart Association; ESC, European Society of Cardiology; AATS, American Association for Thoracic Surgeons; HFA, Heart Failure Association; EACVI, European Association of Cardiovascular Imaging; EHRA, European Heart Rhythm Association; EAPCI, European Association of Percutaneous Cardiovascular Interventions. TMVr, Transcatheter Mitral Valve Repair; HT, heart Transplantation; VAD, Ventricle Assist Device; CABG, Coronary Artery Bypass Grafting; GDMT, Goal-Directed Medical Therapy; CRT, Cardiac Resynchronization Therapy; HF, Heart Failure.

* COAPT criteria: LVEF 20–50%, LVESD <70 mm, systolic pulmonary pressure <70 mmHg, absence of moderate or severe right ventricular dysfunction or severe TR, absence of hemodynamic instability.

**Table 2.** Inclusion and exclusion criteria of the randomized-controlled trials comparing MV procedure + CBAG versus CABG alone in patients with ischemic SMR.

Studies	CTSN trial severe MR 2014, 2016 [39,43,44]	Bouchard *et al.* 2014 [41]	RIME trial, Chan J, 2012 [35]	Fattouch K *et al*. 2009 [40]
Comparison	MV repair versus MV replacement	MV repair + CABG versus CABG alone	MV repair + CABG versus CABG alone	MV repair + CABG versus CABG alone
Inclusion criteria	Severe ischemic SMR	grade 2+ FIMR with a concomitant need for CABG	CABG and had moderate ischemic MR	CABG + moderate SMR.
	Assessment of mitral regurgitation will be performed using an integrative method.		Definition of moderate ischemic MR: EROA 0.20 to 0.39 cm^2^, RV 30 to 59 mL per beat, RF 30% to 49%, or a vena contracta width of 0.30 to 0.69 cm.	Carpentier’s type IIIb, or Carpentier’s type I, or both
	Eligible for surgical repair and replacement of mitral valve		An integrative approach was used to determine moderate MR.	
	Coronary artery disease with or without the need for coronary revascularization			
Exclusion criteria	Ruptured papillary muscle	Papillary muscle rupture	Papillary muscle rupture	Patients with a recent myocardial infarction (<30 days)
	Planned concomitant Intraoperative procedures (except tricuspid valve repair, patent foramen ovale closure, atrial septal defect closure, or maze procedure)	Concomitant need for aortic valve surgery	LVEF <30%	Unstable hemodynamic status
	Prior mitral valve repair	Life expectancy <12 months from noncardiac causes	Significant aortic valve disease	Concomitant aortic valve operations
	Contraindications to cardiopulmonary bypass	Creatinine >200 mmol/L	Previous or active endocarditis	Organic mitral valve lesions requiring MV replacement
	Clinical signs of cardiogenic shock at the time of randomization Treatment with chronic intravenous inotropic therapy at the time of randomization	SMR > grade 2	NYHA class IV symptoms	Requirement for surgical left ventricular restoration
	Severe irreversible pulmonary hypertension		Unstable angina	
	Myocardial infarction within 7 days		Acute pulmonary edema or cardiogenic shock	
	Congenital heart disease		Significant comorbidities (severe renal impairment, liver impairment, chronic obstructive airways disease)	
	Chronic creatine >2.5 or chronic renal replacement therapy		Other associated conditions that significantly increase the risk of surgery	
	Evidence of cirrhosis or hepatic synthetic failure		Previous cardiac surgery	
	Excessive surgical risk (in the judgment of the surgical investigator)			

**Table 3.** “Holistic” approach of the assessment of patients with heart failure and secondary mitral regurgitation.

	Assessment modalities	Parameters	Favorable criteria for surgery and MV repair in SMR
Patient-level	Clinical assessment	Symptoms	Highly symptomatic (NYHA class II–IV)
		Signs and history of HF	No long history of HF
		Health-related quality of life	Impaired HRQOL because of SMR
		Comorbidities	Life expectancy >1 year
		Ongoing medication — GDMT optimized	Optimized GDMT
		CRT if indicated	
		Frailty assessment	No sign of frailty
		Surgical risk evaluation	Low-intermediate surgical risk
		End-organ assessment	No end-organ dysfunction
Heart-level	EKG ’ Holter	Atrial fibrillation	
	echocardiography	Etiology of HF	Ischemic cardiomyopathy
		LV wall motion anomalies	Absence of aneurysm/dyskinesis
		LVEF	LVEF ≥20%
		LV diameters	LVEDD <65 mm, LVESD <70 mm
		LV volumes	LVESV < 145 mL
		Systolic sphericity index	≤0.7
		RV function	Absence of severe RV dysfunction and severe TR
		Left atrium dimension	Mild left atrial dilatation
		Interpapillary muscle distance	<20 mm
		Other structural heart anomalies	
	Coronary angiogram	CAD	Good vessels targets, especially in the posterolateral wall
	Right heart catheterism	CO, CI, PVR	CI >2 L/min/m^2^
		PAPs	PAPs <70 mm Hg
	Myocardial viability assessment CMR	Scare in the papillary muscle area or posterolateral wall	Presence of both viability and ischemia in the posterolateral wall
Mitral valve-level	2D and 3D TEE or TTE CMR	EROA	ERO ≥30 mm^2^
		Mitral annulus diameter	MV annulus diameter ≤37 mm^2^
		Regurgitant fraction	RF >50%
		Regurgitant volume	RV >60 mL
		Vena Contracta	VC >7 mm
		Tethering area	Tethering area ≤16 mm^2^
		Posterior leaflet angulation	≤45
		Coaptation distance	≤10 mm
		Posterior leaflet tethering distance	≤40 mm
		Leaflets prolapse	Present

CRT, Cardiac Resynchronization Therapy; NYHA, New York Heart Association; LV, left ventricular, LVEF, left ventricular ejection fraction; LVEDD, left ventricular end-diastolic diameter; LVESD, left ventricular end-systolic diameter; LVESV, left ventricular end-systolic volume; CAD, coronary artery disease; EROA, effective regurgitant orifice area; HRQOL, Health-related quality of life; RV, right ventricular function; CO, cardiac output; CI, cardiac index; PVR, pulmonary vascular resistance.
